# Preventive Effect of Epigallocatechin-3-Gallate on Postoperative Cognitive Dysfunction in Aged Rats via Modulation of Microglial Differentiation: An Experimental Animal Study

**DOI:** 10.3390/ijms262311326

**Published:** 2025-11-23

**Authors:** Seung-Wan Hong, Liyun Piao, Eun-Hwa Cho, Eun-Hye Seo, Seong-Hyop Kim

**Affiliations:** 1 Department of Anesthesiology and Pain Medicine, Konkuk University Medical Center, Konkuk University School of Medicine, Seoul 05030, Republic of Korea; 20150077@kuh.ac.kr; 2 Department of Infection and Immunology, Konkuk University School of Medicine, Seoul 05030, Republic of Korea; 3 Korea mRNA Vaccine Initiative, Gachon University School of Medicine, Incheon 13120, Republic of Korea; 4 Department of Medicine, Institute of Biomedical Science and Technology, Konkuk University School of Medicine, Seoul 05030, Republic of Korea

**Keywords:** flavonoid, postoperative cognitive dysfunction, microglia, general anesthesia, neuroinflammation

## Abstract

This study evaluated the effect of epigallocatechin-3-gallate (EGCG), a flavonoid, on postoperative cognitive dysfunction by modulating microglial phenotype expression in aged rats following general anesthesia with isoflurane. Eighteen-month-old male Sprague–Dawley rats were randomly assigned to the EGCG and control groups. EGCG in distilled water (DW) or DW alone was administered orally for 7 days before anesthesia. After anesthesia, cognitive function was assessed using the Y-maze test, and neuronal damage was evaluated histologically. Microglial activation and phenotype differentiation were also analyzed. At 24 h after anesthesia, the alternation ratio was significantly higher in the EGCG group than in the control group (70.00 ± 7.98% vs. 28.14 ± 11.52%, *p* < 0.001). The EGCG group exhibited reduced neuronal damage and microglial activation. Additionally, M1 phenotype activation was significantly lower (30.03 ± 7.73% vs. 51.00 ± 9.83%, *p* < 0.001), and M2 phenotype activation was significantly higher (17.61 ± 5.52% vs. 5.99 ± 2.46%, *p* < 0.001) in the EGCG group than in the control group. In summary, pre-anesthetic administration of EGCG modulated microglial phenotype differentiation, reduced neuronal damage, and improved postoperative cognitive function in aged rats following general anesthesia.

## 1. Introduction

As life expectancy has increased and medical technology has advanced, the likelihood of elderly patients undergoing procedures under general anesthesia has also grown. While many adverse events following general anesthesia are preventable and manageable, some complications, such as postoperative cognitive dysfunction (POCD), can lead to severe, even catastrophic, outcomes. POCD is defined as a decline in cognitive function after anesthesia and surgery [1], and while often transient can persist for several years, resulting in reduced quality of life, loss of functional independence, and increased mortality in vulnerable populations. Elderly patients are particularly susceptible to POCD, and its occurrence can impair recovery after anesthesia and surgery, imposing a significant socioeconomic burden [2,3].

Although the exact mechanisms of POCD remain unclear, it is consistently associated with neuroinflammation, a hallmark also observed in neurodegenerative disorders such as Alzheimer’s disease and Parkinson’s disease [4]. During neuroinflammation in the brain, microglia act as a crucial defense barrier, protecting neurons. These cells can polarize into two distinct phenotypes: pro-inflammatory (M1) and anti-inflammatory (M2) [5].

Flavonoids are polyphenolic secondary metabolites found in plants and fungi that are widely recognized for their neuroprotective properties and their potential in preventing or managing neurodegenerative disorders [6,7,8]. These compounds downregulate microglial activation and attenuate cytokine release in neurodegenerative conditions, thereby exerting significant anti-inflammatory effects. Given the central role of microglial modulation in the pathogenesis of POCD, flavonoids are anticipated to exert beneficial effects on POCD by influencing the balance between the two microglial phenotypes. However, the specific effects of flavonoids on microglia in the context of POCD have not been thoroughly investigated.

Epigallocatechin-3-gallate (EGCG), a major green tea catechin, has been shown to attenuate neuroinflammation through multiple mechanisms, including suppression of pro-inflammatory transcriptional programs like nuclear factor κB (NF-κB)-dependent cytokine expression [9,10], activation of endogenous antioxidant responses [10,11], and modulation of mitogen-activated protein kinase (MAPK) signaling [12]. These observations may offer preliminary rationale to evaluate EGCG as a potential prophylactic strategy against POCD.

We hypothesized that EGCG might prevent POCD by modulating microglial phenotypes. Therefore, we evaluated the effects of EGCG on POCD and its associated modulation of microglial phenotype expression in aged rats following general anesthesia with isoflurane.

## 2. Results

Fourteen rats were enrolled in this study and evenly allocated into the two groups, with no dropouts recorded.

**Figure 1:**
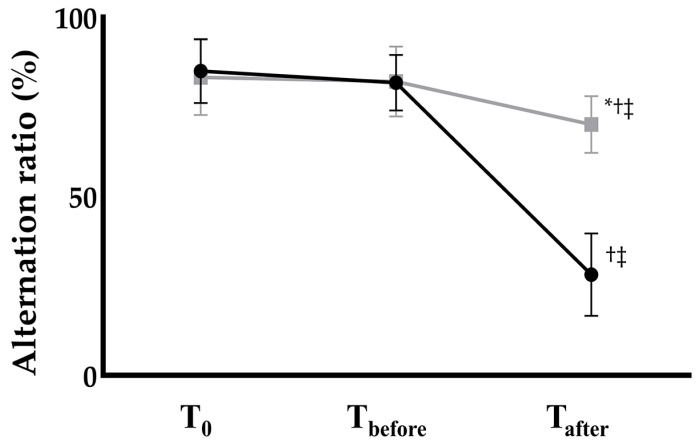
Postoperative cognitive function assessed by the Y-maze alternation ratio in Control (black circles) and EGCG (gray squares) groups. Anesthesia: isoflurane 1.5 vol% for 4 h (endotracheal intubation, controlled ventilation). * *p* < 0.05 vs. Control group; ^†^ *p* < 0.05 vs. T_0_ within the same group; ^‡^ *p* < 0.05 vs. T_before_ within the same group. Abbreviations: T_0_, baseline; T_before_, 1 h before anesthesia; T_after_, 24 h after anesthesia.

The Y-maze test revealed no significant inter- or intra-group differences between the groups at baseline (T_0_) or at 1 h before anesthesia induction (T_before_). However, the alternation ratio measured 24 h after anesthesia induction (T_after_) showed a significant difference between the groups (Figure 1). In the control group, the alternation ratio decreased markedly from 79.70 ± 7.94% at T_before_ to 28.14 ± 11.52% at T_after_, representing a 67% reduction (*p* < 0.001). In contrast, the EGCG group exhibited only a modest but statistically significant decrease from 82.29 ± 8.86% to 70.00 ± 7.98%, corresponding to an approximately 15% reduction (*p* = 0.041)

**Figure 2:**
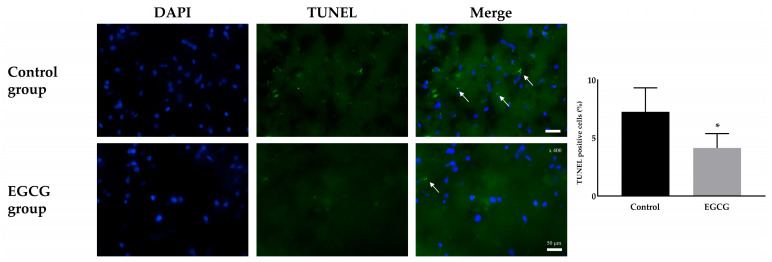
Terminal deoxynucleotidyl transferase (TdT)-mediated deoxyuridine triphosphate (dUTP) nick-end labeling (TUNEL) staining for the damage of neurons. Colors: DAPI (blue, nuclei); TUNEL positive cells (green). White arrows indicate the target cells. Tissue collection: brains collected 24 h after anesthesia; anesthesia: isoflurane 1.5 vol% for 4 h (endotracheal intubation, controlled ventilation). * *p* < 0.05 vs. Control group. Abbreviations: DAPI, 4′,6-diamidino-2-phenylindole staining; Merged, merged image of DAPI staining and TUNEL staining.

Terminal deoxynucleotidyl transferase (TdT)-mediated deoxyuridine triphosphate (dUTP) nick-end labeling (TUNEL) staining demonstrated a significant difference in neuronal damage between the groups. The EGCG group exhibited markedly fewer TUNEL-positive cells than the control group (7.26 ± 2.06% and 4.13 ± 1.24% in the control and EGCG groups, respectively, *p* = 0.007) (Figure 2).

**Figure 3:**
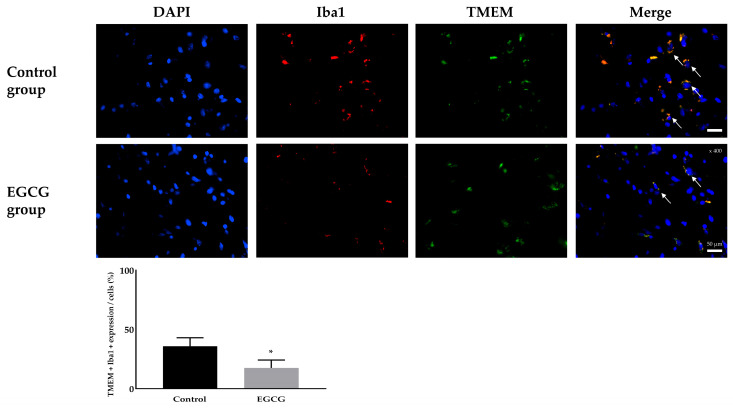
Immunofluorescence staining to detect the activated microglia. Colors: DAPI (blue, nuclei); microglial expression (yellow). White arrows indicate the target cells. Tissue collection: brains collected 24 h after anesthesia; anesthesia: isoflurane 1.5 vol% for 4 h (endotracheal intubation, controlled ventilation). * *p* < 0.05 vs. Control group. Abbreviations: DAPI, 4′,6-diamidino-2-phenylindole staining; Iba1, ionized calcium-binding adapter molecule 1 staining; TMEM119, rabbit transmembrane protein 119 staining; Merged, merged image of DAPI staining, Iba1 staining and TMEM119 staining.

Immunofluorescence staining for activated microglia also showed significant differences. The control group demonstrated a significantly higher expression of activated microglia (35.89 ± 7.24% and 17.50 ± 6.83% in the control and EGCG groups, respectively, *p* < 0.001) (Figure 3).

**Figure 4:**
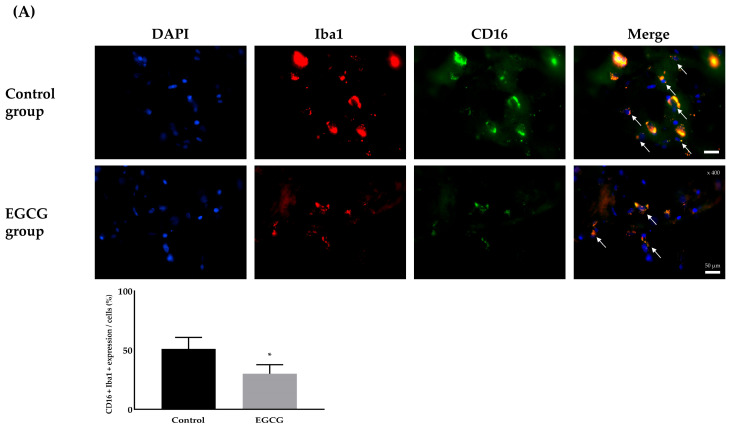

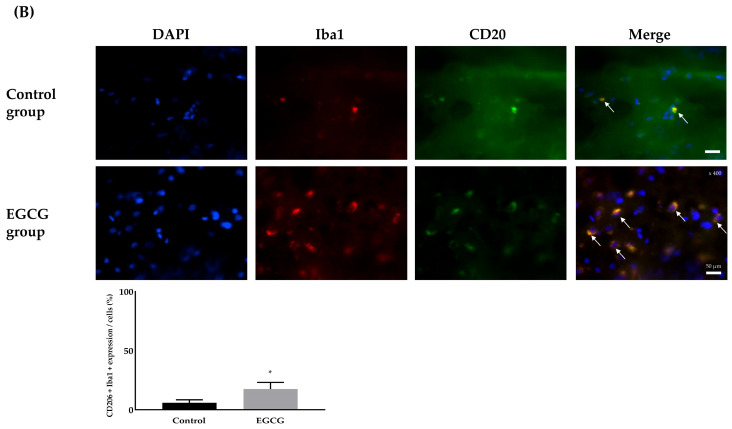
Differentiation for the phenotype expression of microglia, M1 phenotype as a pro-inflammatory producer (**A**) and M2 phenotype as an anti-inflammatory producer (**B**). Colors: DAPI (blue, nuclei); microglial expression (yellow). White arrows indicate the target cells. Tissue collection: brains collected 24 h after anesthesia; anesthesia: isoflurane 1.5 vol% for 4 h (endotracheal intubation, controlled ventilation). * *p* < 0.05 vs. Control group. Abbreviations: DAPI, 4′,6-diamidino-2-phenylindole staining; Iba1, ionized calcium-binding adapter molecule 1 staining; CD, cluster of differentiation; Merged, merged image of DAPI staining, Iba1 staining, and CD16 (for M1 phenotype) or CD206 staining (for M2 phenotype).

Analysis of microglial phenotype differentiation revealed significant differences in the expression of M1 and M2 phenotypes between the groups. The EGCG group showed a significantly lower expression of the M1 phenotype (51.00 ± 9.83% and 30.03 ± 7.73% in the control and EGCG groups, respectively, *p* < 0.001) and a significantly higher expression of the M2 phenotype (5.99 ± 2.46% and 17.61 ± 5.52% in the control and EGCG groups, respectively, *p* < 0.001) (Figure 4).

The pro-inflammatory cytokines TNF-α and IL-1β were significantly lower in the EGCG group than in the control group, whereas the anti-inflammatory cytokines IL-10 and IL-4 were significantly higher in the EGCG group than in the control group (Table 1).

## 3. Discussion

The present study clearly demonstrated that EGCG administered prior to general anesthesia attenuated early anesthesia-related cognitive decline in the Y-maze and mitigated neuronal injury in aged rats exposed to isoflurane. These neuroprotective effects were accompanied by the inhibition of microglial activation and significant modulation of microglial phenotype expression, characterized by reduced expression of the pro-inflammatory M1 phenotype and increased expression of the anti-inflammatory M2 phenotype following EGCG treatment. These findings suggest that EGCG may act as a neuroprotective agent against POCD by directly modulating neuroinflammation.

Based on the convergent readouts in this study, we propose a working model in which EGCG lowers brain cytokine levels, biases microglial polarization toward an anti-inflammatory state, and thereby limits bystander neuronal injury, consistent with the improvement in Y-maze performance at 24 h. In line with prior literature, these effects are plausibly mediated by downregulation of NF-κB-dependent transcription and activation of nuclear factor erythroid 2-related factor 2 (Nrf2)-regulated antioxidant programs, with possible MAPK modulation [9,10,11,12]. However, these pathways were not directly quantified here and require targeted assays in future work.

A variety of pharmacological interventions, including dexmedetomidine, ketamine, dexamethasone, and lidocaine, have been investigated for POCD prevention through targeting neuroinflammatory pathways, with some showing favorable results [13,14,15,16]. Among these, perioperative dexmedetomidine administration has been associated with a reduced incidence of POCD in both clinical and preclinical studies [17,18,19]. However, the perioperative use of such agents requires prescription and monitoring by qualified medical personnel under specific conditions. Consequently, dietary-based strategies for POCD prevention may represent a more accessible and potentially cost-effective alternative. For example, dietary vitamin B12, which is found in foods such as meat, fish, and clams, has been examined in this context. Sha et al. [20] reported that intramuscular vitamin B12 administration attenuated POCD in a rat model. Nevertheless, vitamin B12 supplementation carries certain risks. High vitamin B12 intake may increase the risk of cardiovascular disease [21]. A meta-analysis of cohort studies indicated that supraphysiological serum vitamin B12 concentrations (>600 pmol/L) were associated with increased cardiovascular mortality, particularly in elderly populations [22]. Given the high prevalence of cardiovascular disease with advancing age, vitamin B12 supplementation for POCD prevention should be approached with caution and may not be appropriate for older individuals.

The neuroprotective effects of flavonoids, including EGCG, have been well documented. Silva et al. [23] demonstrated that flavonoids promote microglial polarization toward the M2 phenotype. Almeida et al. [24] reported that flavonoids modulate microglial activity and cytokine release, facilitating remyelination and repair in neurodegenerative diseases. Similarly, Khan et al. [25] found that flavonoid administration reduced the levels of pro-inflammatory cytokines such as IL-6, IL-1β, and TNF-α in a Parkinson’s disease model. The present findings were consistent with these reports, showing that EGCG effectively shifts microglia from a pro-inflammatory M1 phenotype to an anti-inflammatory M2 phenotype in the setting of POCD.

Although excessive flavonoid exposure may theoretically carry mutagenic or genotoxic potential [26], such adverse effects are generally not observed with dietary intake, as achieving plasma concentrations sufficient to elicit toxicity is unlikely [26]. Given its abundance in foods such as parsley, onions, berries, tea, and bananas, dietary EGCG represents a promising and safe alternative to vitamin B12 for POCD prevention in elderly individuals.

In this study, Y-maze testing was performed at three time points: before group allocation, 1 h prior to anesthesia induction, and 24 h after anesthesia. The similar alternation ratios observed at baseline and 1 h before anesthesia suggest that EGCG administration did not affect baseline cognitive function prior to anesthetic exposure. The marked decline in alternation ratio in the control group 24 h after anesthesia, together with the present study’s TUNEL staining and ELISA findings, indicated that a 4 h exposure to general anesthesia in 18-month-old male SD rats induced substantial structural and functional brain damage lasting beyond the immediate perioperative period. However, this result should not be interpreted as evidence that anesthesia is intrinsically harmful. While hemodynamic and respiratory parameters were carefully monitored in the present study, perioperative factors such as transient hypotension or hypoxia, rather than the anesthetic agent itself, may contribute to POCD. Although the impact of such confounders is expected to be similar across groups, their potential influence warrants consideration.

With respect to safety, our preventive regimen (oral EGCG 10 mg/kg/day for 7 days) corresponds by standard body surface area allometric conversion to a human-equivalent dose of approximately 1.6 mg/kg (≈100–110 mg/day for a 60–70 kg adult). This exposure lies well below supplement intakes ≥ 800 mg/day at which elevations in serum transaminases have been reported in interventional studies of green tea catechins [27]. Preclinical toxicology likewise suggests a wide margin: in rats, no observed adverse effect level values for EGCG ranging from 242 to 500 mg/kg/day (depending on matrix and schedule) have been described, whereas hepatotoxicity is more likely with very high oral bolus doses, parenteral administration, or under fasting conditions that increase catechin bioavailability [27,28]. In our study, conducted under non-fasted conditions, no mortality or overt clinical distress was observed during dosing. Nonetheless, hepatotoxicity has been documented in humans using concentrated green tea extracts [29]. Therefore, any clinical translation should employ administration with food, avoid high bolus dosing, and incorporate routine liver enzyme monitoring, particularly in individuals with pre-existing liver disease or polypharmacy.

This study had several limitations. First, it was conducted in a rat model, and direct translation of these findings to human patients will require further clinical studies. Second, we selected the 24 h time point to capture early anesthesia-related cognitive decline while minimizing recovery-phase confounders. This timing aligns with the temporal dynamics of the present study’s mechanistic endpoints, microglial phenotype markers and cytokines which show their clearest changes in the immediate post-anesthetic period [30]. Accordingly, we interpret the present findings strictly within this early window and do not infer durability beyond 24 h. Third, the present study did not include pathway-level measurements to confirm the putative mechanism. We did not assess NF-κB activation, Nrf2 signaling, or MAPK readouts thus, the mechanistic interpretation remains inference-based, and future studies will incorporate these assays and perturbation approaches to establish causality. Fourth, the present design did not include EGCG-only or naive (no treatment/no anesthesia) comparators, which constrains external validity and precludes dissociating anesthesia-independent effects of EGCG on microglial phenotype and cytokines. Future work will employ factorial designs to establish generalizability and to determine interaction with anesthetic exposure. Finally, the interventions were restricted to a single flavonoid (EGCG) and a single anesthetic (isoflurane) to reduce heterogeneity and preserve power; this limits external validity and prevents evaluation of dose–response and compound-by-anesthetic interactions. Because isoflurane is a volatile anesthetic with neuroimmune signatures that differ from those of other volatile agents and from intravenous anesthetics, the observed effects may be volatile-agent specific and should not be generalized to all anesthetic regimens. Future work using multi-arm or factorial designs will be required to establish generalizability across flavonoids and anesthetic classes.

In conclusion, pre-anesthetic administration of EGCG effectively modulated microglial phenotypes and reduced neuronal damage in the brains of aged rats following general anesthesia. These results suggest that EGCG has considerable potential as a preventive strategy for POCD in elderly individuals, likely mediated through the amelioration of neuroinflammation.

## 4. Materials and Methods

All experiments were conducted in accordance with the National Institutes of Health guidelines for the care and use of laboratory animals. Approval was obtained from the Institutional Animal Care and Use Committee (IACUC) of Konkuk University (No. KU21013, on 1 July 2021), and all procedures were performed at the Konkuk University Laboratory Animal Research Center in compliance with IACUC guidelines. The reporting of this study complies with the ARRIVE guidelines for animal research. Manufacturer and catalog numbers are reported for all antibodies and dyes; for legacy experiments conducted during July–August 2021, historical lot numbers were not recoverable from laboratory records.

### 4.1. Animal Preparation and Grouping

Eighteen-month-old male Sprague–Dawley rats were purchased from Orient Bio (Seongnam, Republic of Korea). The rats were housed in cages with free access to water and food. The animal room was maintained under a standard 12 h light/dark cycle (lights on at 07:00 and off at 19:00) at a temperature of 25 °C [31]. All rats were acclimated to the experimental conditions for 7 days prior to the study [32] and were clinically healthy at arrival and after the 7 days acclimation period.

Following acclimation, the rats were randomly assigned to EGCG and control groups. Rats in the EGCG group received oral EGCG (10 mg/kg; Sigma-Aldrich, St. Louis, MO, USA) dissolved in 0.8 mL distilled water (DW). Rats in the control group received the same volume of DW [33]. Treatments were administered once daily at 10:00 for 7 consecutive days. The final dose was given 24 h before anesthesia, and no EGCG was administered at induction.

Cognitive testing was scheduled at three time points: baseline (T_0_), 1 h prior to anesthesia induction (T_before_), and 24 h after anesthesia (T_after_). Prior to group allocation, all rats underwent the Y-maze test to rule out pre-existing cognitive dysfunction. Any rat exhibiting cognitive impairment, defined as an alternation ratio < 40%, on the day following the 7-day acclimation period and before group allocation was excluded from the study. All tests were performed at the same time of day to minimize circadian variability.

### 4.2. Establishment of the POCD Model After General Anesthesia with Isoflurane

After completing the 7-day pre-anesthetic regimen (no EGCG or DW was given on the day of anesthesia), general anesthesia with isoflurane without any surgical incision (Hana Pharm, Seoul, Republic of Korea) was induced in both groups to establish the POCD model [34,35]. Anesthesia induction was performed via intraperitoneal injection of ketamine (80 mg/kg; Ketamine 50, Yuhan, Seoul, Republic of Korea) to enable atraumatic endotracheal intubation and stable initial immobilization. Anesthesia maintenance was performed with isoflurane alone. A heating pad (Hanil Electric Co., Ltd., Seoul, Republic of Korea) was placed on the surgical platform to maintain body temperature at approximately 37 °C throughout the procedure.

Each rat was placed in supine position and secured on the surgical platform for endotracheal intubation. A 16 G catheter (Dukwoo Medical, Hwaseong, Republic of Korea) was inserted through the larynx into the trachea, and correct placement was confirmed by symmetrical chest expansion. The catheter was then connected to a ventilator (Harvard Apparatus, Holliston, MA, USA) with the following settings: fraction of inspired oxygen (FiO_2_), 1.0; inspired flow rate, 0.24 L/min; tidal volume, 6 mL/kg; respiratory rate, 50 breaths/min; inspiration-to-expiration ratio, 1:1; and positive end-expiratory pressure, 5 cmH_2_O. Endotracheal intubation with controlled ventilation was chosen over a facemask to precisely deliver a constant isoflurane concentration and to maintain normoventilation and oxygenation during the 4 h exposure, reducing variability from leaks, dead-space effects, and hypoventilation/hypercapnia.

The ventilator settings were maintained for 4 h, during which 1.5 vol% isoflurane was administered via the endotracheal catheter. After 4 h, the isoflurane vaporizer was turned off, and mechanical ventilation continued until spontaneous respiration was fully restored. Once spontaneous ventilation was confirmed, the endotracheal catheter was removed. Rats were observed on the surgical platform until complete recovery from anesthesia, after which they were returned to their cages.

### 4.3. Assessment of Cognitive Function with Y-Maze Test

The Y-maze apparatus (Ugo Basile, Gemonio, Italy) consisted of three arms labeled A, B, and C. Each arm measured 50 cm in length, 15 cm in width, and 30 cm in height, and the arms were connected at 120° angles to form an equilateral triangle. At the start of the test, arm C was blocked with a board placed at the center of the apparatus, allowing movement only between arms A and B. Each rat was placed at the end of arm A and allowed to freely explore arms A and B for 10 min for acclimation to the apparatus. After acclimation, the barrier blocking arm C was removed, and the rat was allowed to explore all three arms freely. Movements were recorded with a video camera for 5 min.

The total number of entries into each arm and the number of spontaneous alternations were recorded. The alternation ratio was calculated using the following formula: [(Number of spontaneous alternations)/(Total arm entries − 2)] × 100%.

### 4.4. Brain Tissue Preparation

Following the Y-maze test at 24 h after anesthesia, each rat was anesthetized in an induction chamber with 5 vol% isoflurane in a mixture of oxygen (0.3 L/min) and nitrous oxide (0.7 L/min) and subsequently sacrificed. The abdominal cavity was opened to expose the abdominal aorta, and 1× phosphate-buffered saline (PBS) was perfused through the aorta until complete exsanguination was achieved. The brain, including the cerebral cortex and hippocampus, was dissected and collected.

The brain was divided into two hemispheres. The left hemisphere was used to assess neuronal damage and microglial activation with phenotype differentiation. This hemisphere was embedded in a cell culture plate containing optimal cutting temperature compound and stored in a deep freezer (Thermo Fisher Scientific, Waltham, MA, USA) until analysis. The right hemisphere was used to assess the degree of inflammation. This hemisphere was placed in a 5 mL Eppendorf tube (Eppendorf, Hamburg, Germany) and stored in a deep freezer until further processing.

Neuronal damage was evaluated using TUNEL staining. Microglial activation and phenotype differentiation were assessed using immunofluorescence staining. The degree of inflammation was determined by enzyme-linked immunosorbent assay (ELISA) for cytokine detection.

### 4.5. TUNEL Staining for Neuronal Damage

The frozen brain hemisphere was mounted on a saline-coated slide and sectioned to a thickness of 20 μm using a cryotome (Leica Microsystems, Wetzlar, Germany). The slide was immersed in 4% paraformaldehyde in 1× PBS for 15 min and then washed with 1× PBS for 5 min, three times. The immersed and washed slide was permeabilized with proteinase K solution (20 μg/mL) and incubated for 40 min. After permeabilization and incubation, the slide was immersed again in 1× PBS for 5 min. The tissue on the slide was again fixed with 4% paraformaldehyde for 5 min and washed with 1× PBS for 5 min. Following fixation and washing, the tissue was equilibrated with 100 μL of equilibration buffer for 5 min at room temperature and subsequently stained with TdT solution, which consisted of 45 μL equilibration buffer, 5 μL nucleotide mix, and 1 μL TdT from the TUNEL staining kit; the mixture was allowed to react with the tissue. The section was immersed in TdT solution for 60 min at 37 °C in a humidified chamber, followed by immersion in 2× saline sodium citrate buffer from the TUNEL staining kit to stop the reaction. The tissue was then washed with 1× PBS for 5 min, three times. Counterstaining was performed with 4′,6-diamidino-2-phenylindole (DAPI; 0.5 μg/mL in 1× PBS, Invitrogen, Carlsbad, CA, USA, cat D21490) for 5 min, and the tissue was mounted with antifade medium (Vector Laboratories, Newark, CA, USA) and covered with a coverslip. Neuronal damage was evaluated and analyzed using confocal fluorescence microscopy (Nikon Corporation, Tokyo, Japan).

### 4.6. Immunofluorescence Staining to Detect Activated Microglia

The frozen brain hemisphere was mounted on a saline-coated slide and sectioned to a thickness of 20 μm with a cryotome (Leica Microsystems, Wetzlar, Germany). Activated microglia were detected using double immunofluorescence staining. The specific microglial marker transmembrane protein 119 (TMEM119) antibody (Synaptic Systems, Goettingen, Germany, cat 400-003) using activated microglial marker ionized calcium-binding adapter molecule 1 (Iba1) antibody (Invitrogen, Carlsbad, CA, USA, cat MA5-27726). Before staining, tissue mounted on the saline-coated slide was warmed to room temperature for 30 min and then fixed in 4% paraformaldehyde for 10 min. The warmed tissue was incubated with 5% bovine serum albumin (BSA) for 1 h at room temperature to block non-specific binding. After blocking, the tissue was washed with 1× PBS for 5 min, three times, and then incubated with a mixture of TMEM119 and Iba1 as primary antibodies in 1× PBS containing 1% BSA for 1 h in the dark. Next, the tissue was washed with 1× PBS for 5 min, three times, and incubated with a mixture of rabbit Alexa Fluor 488 (Thermo Fisher Scientific, Waltham, MA, USA, cat A-11094) and mouse Alexa Fluor 594 (Thermo Fisher Scientific, Waltham, MA, USA, cat A17045) as secondary antibodies in 1× PBS with 1% BSA for 1 h at room temperature in the dark. Next, the tissue was washed with 1× PBS for 5 min, three times. The washed tissue was incubated with DAPI for nuclear staining for 5 min and again washed with 1× PBS for 5 min, three times. After the staining processes were completed, the tissue was mounted with a coverslip using a drop of antifade mounting medium, and the coverslip was sealed with nail polish to prevent drying and movement under the microscope. Rabbit immunoglobulin G (Bioss Antibodies, Woburn, MA, USA, cat bs-0295P) and mouse immunoglobulin G (Thermo Fisher Scientific, Waltham, MA, USA, cat 88-50420-22) were used as negative controls in place of the primary antibodies. The stained tissue sections were observed under a fluorescence microscope (Olympus, Tokyo, Japan).

### 4.7. Differentiation of M1 and M2 Microglial Phenotype Expression

The frozen brain hemisphere was mounted on a saline-coated slide and sectioned to a thickness of 20 μm using a cryotome (Leica Microsystems, Wetzlar, Germany). The differentiation of microglial phenotype expression into M1 and M2 types was conducted using double immunofluorescence staining. Cluster of differentiation 16 (CD16) antibody (Bioss Antibodies, Woburn, MA, USA, cat bs-6028R) for the M1 phenotype and CD206 antibody (Bioss Antibodies, cat bsm-60761R) for the M2 phenotype were used for the detection of differentiation. The staining process followed the same procedures described for immunofluorescence staining to detect activated microglia. The mixture of CD16 and Iba1 was used as the primary antibodies for M1 phenotype detection, and the mixture of CD206 and Iba1 was used for M2 phenotype detection. The mixture of rabbit Alexa Fluor 488 (Thermo Fisher Scientific, Waltham, MA, USA) and mouse Alexa Fluor 594 (Thermo Fisher Scientific, Waltham, MA, USA) was used for the secondary antibodies in both cases. Rabbit immunoglobulin G (Bioss Antibodies) and mouse immunoglobulin G (Thermo Fisher Scientific, Waltham, MA, USA) were used as negative controls in place of the primary antibodies. The stained tissues were examined under a fluorescence microscope (Olympus).

### 4.8. ELISA for Cytokine Detection

The pro-inflammatory cytokines tumor necrosis factor-α (TNF-α) (Abcam, Cambridge, UK, cat ab100785) and interleukin-1β (IL-1β) (Abcam, Cambridge, UK, cat ab100768), along with the anti-inflammatory cytokines IL-10 (Abcam, Cambridge, UK, cat ab100765) and IL-4 (Abcam, Cambridge, UK, cat ab100770), were measured. The frozen whole-brain right hemisphere was homogenized and centrifuged at 12,000× *g* for 15 min at 4 °C. The supernatant was collected and used for cytokine quantification using ELISA kits. Cytokine levels were determined using a microplate reader (Molecular Devices, San Jose, CA, USA). For each sample, analyte concentrations from ELISA (pg/mL, dilution-corrected) were normalized to the total protein concentration (mg/mL) measured by the bicinchoninic acid assay and expressed as pg/mg protein. No plasma or serum samples were used.

### 4.9. Statistical Analysis

The primary endpoint was the between-group difference in the change in Y-maze alternation from 1 h pre-anesthesia (T_before_) to 24 h post-anesthesia (T_after_), to evaluate the effect of EGCG on POCD. The secondary outcome was the expression of the M1 phenotype in microglia, used to assess the effect of EGCG on microglial phenotype expression. In a pilot study with three rats per group, the alternation ratio showed a decrease of 20.83 ± 25.00% in the EGCG group and 75.00 ± 28.87% in the control group between T_before_ and T_after_. The expression levels of the M1 phenotype in microglia were 24.73 ± 5.44% in the EGCG group and 57.14 ± 3.06% in the control group. Based on these pilot data, the required sample sizes were calculated using G*Power v3.1.9.7 (Heinrich Heine University Düsseldorf, Düsseldorf, Germany) (two-sample *t*-test, two-sided α = 0.05, power = 0.90, 1:1 allocation), the pooled SD was 27.00%, giving Cohen’s d = (75.00 − 20.83)/27.00 = 2.01. Under these inputs, the required sample sizes were seven for the primary outcome (a total of 14 rats) and secondary endpoints were analyzed with the same group sizes and are interpreted as exploratory.

All statistical analyses were conducted using GraphPad Prism v7.0 software (GraphPad Software, LLC, San Diego, CA, USA). One-way repeated-measures analysis of variance was used for intra-group comparisons across three time points, and unpaired Student’s *t*-tests were applied for inter-group comparisons at each time point. Data are presented as mean ± standard deviation, with significance evaluated at a threshold of *p* < 0.05.

## Figures and Tables

**Table 1:** Enzyme-linked immunosorbent assay (ELISA) for the detection of cytokines between EGCG group and Control group. Tissue collection: brains collected 24 h after anesthesia; anesthesia: isoflurane 1.5 vol% for 4 h (endotracheal intubation, controlled ventilation).

	Control Group	EGCG Group	*p* Value
Pro-inflammatory cytokines			
TNF-α (pg/mg protein)	1490.51 ± 350.76	646.46 ± 395.84	0.001
IL-1β (pg/mg protein)	1464.32 ± 693.60	402.51 ± 414.05	0.006
Anti-inflammatory cytokines			
IL-10 (pg/mg protein)	19.91 ± 3.34	35.41 ± 1.81	<0.001
IL-4 (pg/mg protein)	67.60 ± 43.43	195.39 ± 27.71	<0.001

Data are expressed as mean ± standard deviation. Abbreviations: TNF-α, tumor necrosis factor-α; IL, interleukin.

## Data Availability

The raw data supporting the conclusions of this article will be made available by the authors on request.
